# Air embolism complicating endoscopic retrograde cholangiopancreatography

**DOI:** 10.2144/fsoa-2023-0174

**Published:** 2024-05-24

**Authors:** Mouna Medhioub, Becem Trabelsi, Amal Khsiba, Mohamed Saied, Moufida Mahmoudi, Asma Ben Mohamed, Manel Yacoubi, Lamine Hamzaoui, Mechaal Ben Ali

**Affiliations:** 1 Gastroenterology Department, Mohamed Taher Maamouri Hospital, Mrezgua, 8000, Nabeul, Tunisia; 2 Anesthesia Department, Mohamed Taher Maamouri Hospital, Mrezgua, 8000, Nabeul, Tunisia; 3 Université de Tunis El Manar – Faculté de médecine de Tunis, 1006, Tunisie

**Keywords:** air embolism, cardiac ultrasound, cardiovascular collapse, complication, diagnostics, endoscopic retrograde cholangiopancreatography

## Abstract

**Aim:** Venous air embolism is a rare but potentially life threatening complication of endoscopic retrograde cholangiopancreatography. Diagnosis is difficult because of the lack of specific signs or symptoms. **Case:** A 62-year-old man underwent endoscopic retrograde cholangiopancreatography for choledocholithiasis. A cardiovascular collapse occurred during the procedure. The echocardiography showed air within the right ventricle. Aspiration of air from the right ventricle was done and saved the patient's life. **Conclusion:** We highlight through this case that increased awareness is essential for prompt recognition of the air embolism to allow life-saving therapy.

## Plain language summary

Endoscopic treatment is the first-line treatment for biliary stones. It may be associated with various complications. We report the case of a 62-year-old patient who underwent endoscopic treatment for biliary stone. During the procedure, he experienced disturbed cardiac function due to the passage of air bubbles into the cardiac cavities. The bubbles were aspirated with a good outcome.

## Abstract (plain language summary)

Venous air embolism is a rare complication of endoscopic retrograde cholangiopancreatography.

The diagnosis must be suspected in the presence of unexplained cardiovascular and respiratory signs with a sudden onset.

The diagnosis is based on an early cardiac ultrasound.

Early aspiration of cardiac air bubbles often saves the patient's life.

Aspiration of cardiac air bubbles saves patient's life early cardiac ultrasound.

This case report highlights the importance of endoscopist and anesthetist awareness, which allows for prompt recognition of air embolism and early therapy administration.

Endoscopic retrograde cholangiopancreatography (ERCP) has become an invaluable minimally invasive therapeutic method in the management of many biliary and pancreatic diseases. The most common complications of ERCP are: pancreatitis, bleeding, cholangitis, perforation and sedation-related cardiopulmonary adverse events [1].

Air embolisms are rare, but are a potentially life-threatening complication. Air or CO_2_, used for insufflation, induces a favorable pressure gradient, and the air is admitted into the vascular system through a damaged blood vessel [2]. Air embolisms can be limited to the portal venous system or can evolve into a systemic air embolism, with potentially fatal complications including cardiovascular, pulmonary and neurological damage [3]. The diagnosis may be difficult because its presentation can mimic symptoms related to a patient's comorbidities or sedation-related adverse events [2]. Early recognition and appropriate management of air embolism are essential to reduce morbidity and mortality rates. We report the case of a patient with common bile duct stone who had developed cardiovascular collapse secondary to gas embolism during ERCP.

## Case report

A 62-year-old man, with a medical history of hypertension and cholecystectomy, was admitted for epigastric pain radiating to the back with fever.

Physical examination revealed right upper quadrant and epigastric tenderness on palpation with fever (38.9°C) and jaundice. Laboratory tests showed an increase in transaminase level two-times that of normal; alkaline phosphatase levels up to 5.5-times the upper reference; gammaglutamyl transferase level six-times the upper reference; and bilirubin level to eight-times the upper reference. Lipasemia was within normal range and C-reactive protein level was 138 mg/l.

The abdominal ultrasound revealed a dilatation of the intra and extra hepatic bile ducts with evidence of two stones in the main bile duct, measuring 8 and 10 mm in diameter, respectively. The diagnosis of acute cholangitis was retained. The patient was treated with intravenous antibiotics. He underwent ERCP for stone removal on the third day of hospitalization.

**Figure 1.**
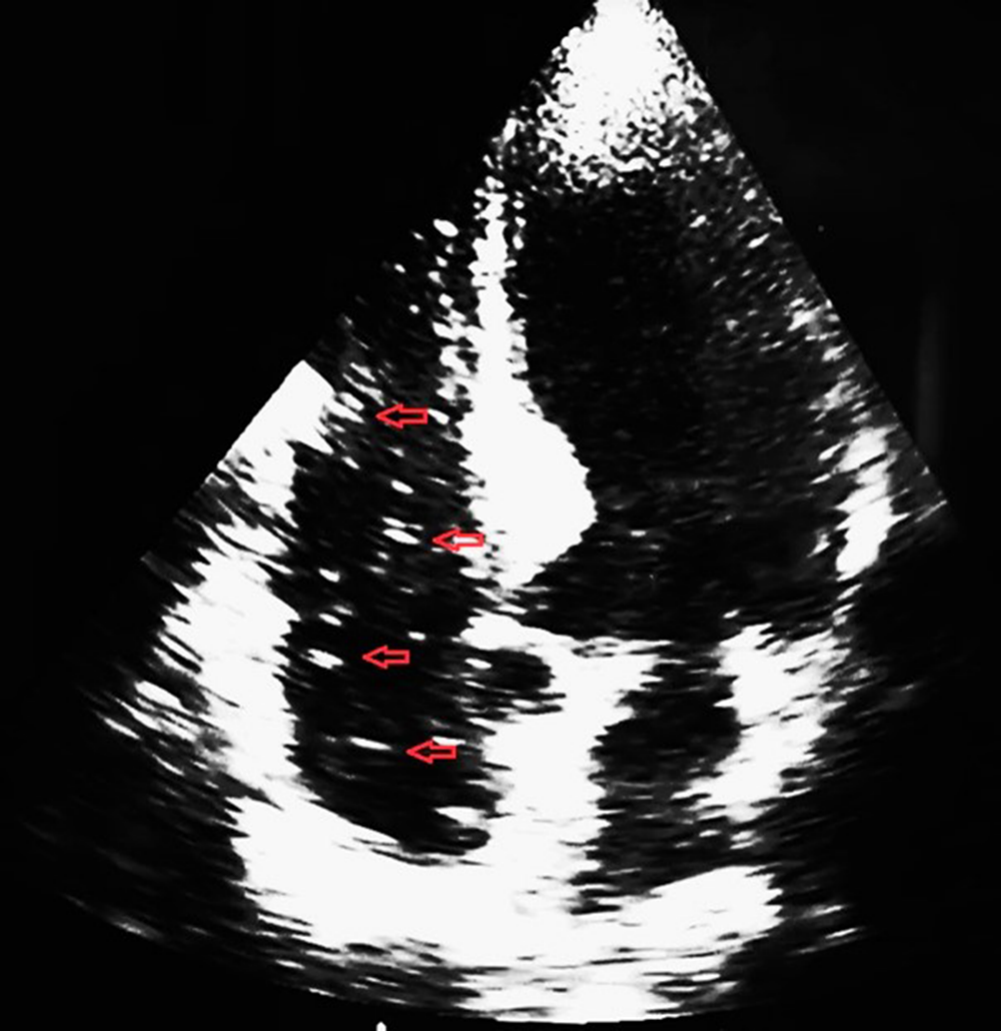
Air bubble in the right ventricle on cardiac ultrasound.

The procedure was performed under general endotracheal anaesthesia. Monitoring included non-invasive arterial blood pressure, pulse oximetry and 5-lead electrocardiography. Patient was in the semi prone position. The endoscopist used air for insufflation. After two papillary cannulation attempts, the selective common bile duct cannulation was successful. Cholangiography showed a dilated extra hepatic bile duct (12 mm) with fill defects consistent with choledocholithiasis. Before performing sphincterotomy, precipitous drop in oxygen saturation (SpO_2_) and blood pressure occurred. The electrocardiogram (ECG) tracing rapidly progressed from normal sinus to deep bradycardia. The procedure was interrupted. The patient was placed in the trendelenburg position and intensive cardiopulmonary resuscitation was immediately started. The patient was admitted to the intensive care unit with blood pressure of 86/56 mmHg, heart rate of 52 bpm and oxygen saturation of 89%. The bedside *trans* thoracic echocardiography (TTE) showed a ‘white-out’ effect of the right heart due to entrapped air in both the atrium and the ventricle with a normal left ventricular function (Figure 1).

A multi orifice central venous catheter was placed in the right atrium through the jugular vein. Aspiration of embolized air was made under TTE image guidance. A cerebral and a chest computed tomography showed no more air embolism. The patient was extubated within 24 h.

## Discussion

Venous air embolism (VAE) can be a serious complication of any endoscopic diagnostic and therapeutic procedures including: ERCP, upper gastro-intestinal endoscopy, enteroscopy, endoscopic ultrasound, colonoscopy [2]. The majority of VAE cases have been related to ERCP. VAE still rare on ERCP, its incidence has been estimated at 2.4% on a prospective cohort study of patients who underwent ERCP and were monitored for VAE with a precordial doppler ultrasound [4]. Only the half of patients with VAE had clinical symptoms suggesting a lower incidence of systemic VAE.

The main risk factors for an air embolism related to ERCP are trauma or inflammation of the bile duct as the case of our patient. The trauma may be caused by: biliary sphincterotomy, stent placement, balloon dilatation, direct cholangioscopy or previous endoscopic manipulations with latent bilio-portal fistulas [2,4–7]. Even high intra-ductal pressure, due to contrast administration or excessive air insufflation, may cause VAE [1].

Indeed, the maximum flow rates of air insufflation may reach 2000 ml/min [8]. This high intraluminal pressure coupled to a physical disruption of a mucosal/vascular barrier, may result in a critical air embolism within very few seconds [8]. For our patient, the mechanisms of VAE were probably multifactorial: inflammation due to acute cholangitis, trauma to the biliary mucosa secondary to multiple attempts to catheterize the biliary tract, and finally intraductal hyperpressure due to air insufflation.

Once in the blood stream, air can pass from the portal vein, through the hepatic veins, into the right ventricle and pulmonary circulation. Air embolism may also reach the arterial system via a patent foramen ovale, a pulmonary arteriovenous shunt or when air volume/debit exceed the pulmonary filtration capacity.

The clinical manifestations depend on the speed and volume of air infused into the blood stream and on the territory affected [2]. They ranged from asymptomatic to highly lethal forms when air embolism concerning the heart and the brain [9,10].

The clinical symptoms of air embolism related to ERCP may be cardiovascular, pulmonary and neurological events [2]. The cardiovascular manifestations can mime anesthetic side effects or acute ischemic symptom's. They may include arrhythmia, hypotension, myocardial ischemia, right heart failure, cardiovascular collapse and/or cardiac arrest [7]. Pulmonary symptoms may include respiratory failure, tachypnea, rales, wheezing and decrease in end-tidal CO_2_ in intubated patients [11]. Early diagnosis requires vigilance and a high index of suspicion. The onset of symptoms, or their worsening on a patient's position, change from prone to supine position, should immediately trigger suspicion for an air embolism [2,7]. Emergency bedside *trans* thoracic and *trans* esophageal echocardiography should be performed, it can confirm the diagnosis by showing air bubbles in the right heart and allows exclusion of the others differential diagnosis [11]. If systemic air embolism is suspected, chest and head computed tomography should be performed to detect the affected organs [1]. If the exploration was delayed, the positive diagnosis could be difficult because the air may be rapidly absorbed from the circulation.

If air embolism is suspected, cardiopulmonary resuscitation measure's should be promptly initiated while the definitive diagnosis is established: 1) interrupting immediately the procedure if at all possible and the endoscopist should decompress the stomach and duodenum on withdrawal to reduce the pressure gradient; 2) endotracheal intubation of the patient with high flow 100% oxygen, which can reduce air bubbles expansion; 3) initiate high volume normal saline infusion to maintain the cardiac output; 4) place the patient in trendelenburg and left lateral decubitus position in order to decrease the spread of air to the cerebral circulation and to force-out air from the right ventricular outflow tract [1,2,7,11].

If air bubbles are detected into the right heart, they can be aspirated via the insertion of a central line as the case of our patient. The specific treatment of air embolism is hyperbaric oxygenation therapy which may reduce air bubble size and increase the oxygen content of arterial blood; this potentially may maintain oxygen supply to ischaemic tissues [2]. Precordial doppler ultrasound monitoring allows the detection of gas embolism at a pre-symptomatic stage in high risk patient [4]. For VAE prevention, the use of CO_2_ insufflation during ERCP is recommended because of its high solubility in blood [1]. CO_2_ may reduce the risk of air embolism, but the risk of life threatening events still persists [12].

## Conclusion

In conclusion, air embolism is a rare but potentially life-threatening ERCP's complication. A high index of suspicion for an air embolism should be maintained for patients with risk factors. Increased endoscopist and anesthetist awareness will allow prompt recognition and life-saving therapy administration.
